# From Metabolism to Longevity: Molecular Mechanisms Underlying Metformin’s Anticancer and Anti-Aging Effects

**DOI:** 10.3390/cimb48030286

**Published:** 2026-03-07

**Authors:** Slavica Vujovic, Svetlana Perovic, Milorad Vlaovic, Andjelka Scepanovic, Stasa Scepanovic

**Affiliations:** 1 Faculty of Natural Sciences and Mathematics, University of Montenegro, George Washington Street, bb, 81000 Podgorica, Montenegro vlaovicmilo@gmail.com (M.V.);; 2 Faculty of Medicine, University of Montenegro, Kruševac bb, 81000 Podgorica, Montenegro; 3 Private Health Centre, Polyclinic Filipovic, Cetinjski Put bb, Master Kvart, 81000 Podgorica, Montenegro; 4 Clinic of Gynecology and Obstetrics, University Clinic Centre of Serbia, Faculty of Medicine, University of Belgrade, 11000 Belgrade, Serbia

**Keywords:** metformin, aging-related pathways, AMPK, anticancer molecular mechanisms, geroprotection, longevity, mTOR

## Abstract

Metformin has stood as the primary clinical tool for type 2 diabetes for decades, yet its potential reach into oncology and gerontology is only now being critically dissected. This review evaluates how metformin might actually pull the levers of cancer progression and biological aging. Evidence from across various models suggests that the drug works by recalibrating cellular energy homeostasis—specifically by triggering AMPK and dampening the mTOR pathway. This signaling shift ripples through downstream processes like autophagy and oxidative stress regulation, theoretically slowing tumor growth and pushing back against cellular senescence. However, our look at the literature from PubMed, Scopus, and Web of Science shows a messy reality where preclinical success often stalls during clinical translation. Even though observational data point toward lower cancer rates in diabetic cohorts, these “wins” are frequently skewed by clinical confounders and inconsistent data. This makes the leap from metabolic control to a broad-spectrum anti-aging or anticancer therapy a point of serious contention. We argue that only large-scale, randomized trials can truly verify if metformin is safe and effective for non-diabetic populations. In the end, untangling these molecular routes is the only way to see if metformin belongs in future oncological or healthy aging strategies. That being said, at least mechanistically, metformin definitely offers potential that warrants such large-scale research.

## 1. Introduction

Metformin remains the clinical gold standard for managing type 2 diabetes mellitus (T2DM). Since its introduction, it has maintained its position as a first-line therapy, primarily valued for its consistent efficacy in lowering HbA1c and its well-established safety profile [1]. However, in recent years, the focus of metformin research has shifted significantly. While its role in glucose suppression is clear, the underlying molecular landscape remains surprisingly complex and, in several aspects, contentious. Beyond the classic inhibition of hepatic gluconeogenesis, it is now evident that metformin’s influence reaches into skeletal muscle and the intestinal environment, involving a sophisticated interplay between AMPK signaling and intestinal receptors [2,3].

This metabolic versatility has sparked interest in “repurposing” metformin for conditions far beyond diabetes—most notably in oncology and gerontology [4,5]. The last twenty years have seen a surge in data exploring whether metformin can actually lower cancer incidence or slow down the biological clocks of aging [6]. At the cellular level, the drug appears to “reprogram” energy sensing. By activating AMPK and suppressing the mTOR pathway, metformin targets the very mechanisms that drive tumor growth and cellular senescence [7,8]. Consequently, it is no longer viewed just as an antidiabetic agent, but as a potential candidate for broad-spectrum metabolic intervention [6,9].

The first real hint of metformin’s anticancer potential emerged in 2001, when a study on hamsters showed that metformin treatment significantly reduced the development of pancreatic lesions [10]. Since that observation, a mountain of epidemiological data has attempted to link T2DM, obesity, and cancer risk. Observational studies frequently suggest that diabetic patients on metformin have lower rates of various malignancies—including colorectal, liver, and breast cancers—compared to those on other treatments [7,11,12].

However, we must approach these findings with caution. When Evans et al. (2005) first reported a reduced cancer risk in a Scottish cohort, it opened the floodgates for similar research [8], yet the results have been far from uniform. While some meta-analyses point to a 31% reduction in tumor incidence [13], other studies on lung and prostate cancers show almost no survival benefit [14,15,16]. For instance, in colorectal cancer, some data sets even failed to show a statistically significant advantage (HR 1.06) [17]. A massive 2023 meta-analysis of 80 studies recently tried to settle this, suggesting that while breast cancer patients might see the most gain, we still lack the definitive evidence that only large-scale randomized controlled trials (RCTs) can provide [18].

The core of the problem lies in the “noise” of clinical data. We cannot simply equate correlation with causation here. Many of the reported benefits might be skewed by clinical confounders; for example, metformin users often have less severe or “younger” diabetes than those prescribed insulin. The situation is further complicated by the common co-prescription of aspirin or statins, which have pleiotropic effects of their own and make it challenging to pinpoint the precise role of metformin. The data’s ongoing heterogeneity is a result of the frequent inconsistencies in the length of diabetes and the precise glycemic control attained (HbA1c levels) between studies. For this reason, we must go beyond clinical observations and investigate the drug’s direct molecular actions in greater detail.

Mechanistically, metformin operates through several distinct routes. In the gut (enterocytes), it appears to disrupt mitochondrial complex I, which shifts the cellular energy balance and triggers AMPK signaling [2,3]. In the liver, however, it can also act independently of AMPK. By noncompetitively blocking mitochondrial glycerol-3-phosphate dehydrogenase (mG3PD), metformin directly shuts down the conversion of lactate and glycerol into glucose [19]. More recently, an epigenetic layer has been identified: metformin can upregulate microRNAs like let-7, which in turn disrupts the TET3–HNF4α axis to suppress hepatic glucose production [20]. This multi-layered mechanism—spanning from mitochondrial enzymes to microRNA regulation—highlights why metformin remains one of the most intriguing molecules in modern pharmacology.

## 2. Materials and Methods

### 2.1. Literature Search and Study Selection

This article was designed as a narrative review of the literature focusing on the molecular mechanisms underlying the anticancer and anti-aging effects of metformin. To ensure transparency and methodological rigor, we employed a structured approach to literature identification and selection.

A literature search was performed using the PubMed, Scopus, and Web of Science databases, covering literature until March 2025. The search terms were combined using the keyword ‘metformin’ and terms related to the key molecular and biological pathways, including AMPK, mTOR signaling, aging, and cancer. Some examples of search terms included “metformin AND AMPK,” “metformin AND mTOR,” “metformin AND aging,” and “metformin AND cancer.”

After combining the search results from all three databases, a total of 3200 articles were found. The duplicates (approximately 1200) were removed before screening. The remaining 2000 articles were screened for relevance to the mechanistic and clinical aspects of the review by title and abstract. At this stage, 1600 articles were removed because they did not have information regarding molecular mechanisms, were not clinically relevant, or were not relevant to the manuscript.

The full-text screening was done for 400 articles. Out of these, 294 articles were excluded because they did not have any mechanistic data, had data related to supra-pharmacologic doses of metformin that are not clinically relevant, or were published in languages other than English.

A total of 106 articles were selected for the qualitative analysis, which is equivalent to the number of references included in the final manuscript.

### 2.2. Data Synthesis

Relevant data were extracted and qualitatively synthesized to identify key molecular pathways influenced by metformin, including AMPK activation, mTOR inhibition, regulation of mitochondrial metabolism, oxidative stress, and cellular senescence. The findings were integrated into a thematic narrative aimed at highlighting shared and divergent mechanisms underlying metformin’s metabolic, anticancer, and anti-aging effects.

## 3. Results and Discussion

This review combines the existing knowledge about the molecular pathways through which metformin works as an anticancer and anti-aging compound. The main crucial biological pathways through which metformin works as an anticancer and anti-aging compound are the activation of AMPK, the inhibition of the mTOR pathway, the regulation of oxidative stress levels, and the modulation of the metabolism of mitochondria. Although the final compound has been found to be useful in the treatment of diabetes, its antiproliferative properties against cancer and its anti-aging effect can be employed in various applications. This will be explained in the later sections.

### 3.1. Metformin-Mediated Inhibition of Mitochondrial Activity, Nuclear Pore Function, and ACAD10 Induction

Here, we focus on the direct, insulin-independent mechanisms of metformin’s anticancer action. Metformin’s effects on tumor cells are multitargeted and can broadly be categorized as direct (insulin-independent) or indirect (insulin-dependent) mechanisms [21]. A key direct target is the mitochondrial respiratory chain. Because cancer cells rely heavily on glucose metabolism (the “Warburg effect”), their elevated glucose uptake can lead to local glucose depletion relative to surrounding normal tissues [22,23].

**Figure 1:**
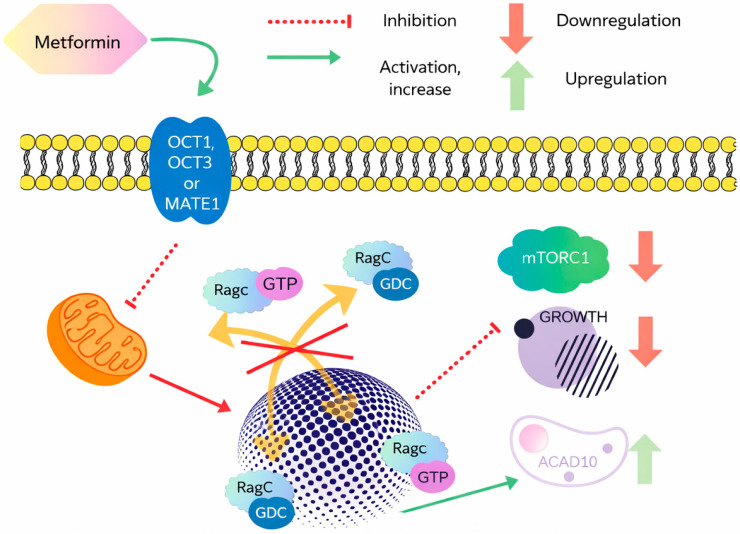
Model of a molecular mechanism of metformin lifespan extending and cell growth inhibition: the blue sphere represents the nucleus. This diagram maps out the way metformin reshapes a cell’s internal signaling to put the brakes on growth. It all starts with the drug’s entry through specific gatekeepers like OCT1, OCT3, or MATE1. Once metformin settles inside, it immediately targets the mitochondria, creating a metabolic ripple effect that alters how the cell functions. A standout feature in this process is the disruption of the Rag GTPase cycle. Usually, the cell relies on a smooth transition of RagC-GDP to its active state to keep things running, but metformin acts as a roadblock here—effectively shown by the red ‘X’ on the map. By cutting off this activation, the drug manages to quiet down mTORC1, which is essentially the cell’s main engine for building proteins and growing. As this growth engine stalls, the cell undergoes a clever metabolic pivot: it ramps up ACAD10 levels. This shift suggests that instead of focusing on expansion, the cell begins to prioritize the efficient breakdown of fatty acids to manage its energy reserves more sustainably.

Wu and colleagues identified two molecular targets linking metformin to antitumor activity within the same pathway: the nuclear pore complex (NPC), responsible for macromolecular trafficking, and acyl-CoA dehydrogenase 10 (ACAD10) [24]. After entering cancer cells via organic cation transporters (OCT1 and OCT3) and the multidrug and tox-in extrusion transporter (MATE1) [25,26], metformin inhibits mitochondrial complex I. This leads to reduced intracellular energy levels, limits transport of the RagA–RagC GTPase heterodimer through the NPC, and ultimately inactivates mTORC1, thereby suppressing tumor cell proliferation (Figure 1). Concurrently, metformin also stimulates the transcriptional upregulation of ACAD10, which regulates β-oxidation and has been shown to extend the longevity of Caenorhabditis elegans [27].

In both human melanoma and pancreatic cancer cells, the restrictions caused by the NPC and the elevation of ACAD10 induced by biguanide drugs have also been noted. However, the abrogation of protective actions through the forced opening of nuclear pores or the genetic ablation of ACAD10 illustrates the importance of functional NPC-ACAD10 signaling in metformin-induced cancer prevention and longevity [27]. Biochemical experiments also showed that the antiproliferative effect in cancer cells at various doses of metformin occurs through the depletion of the TCA cycle due to the reduced activity of the mitochondria complex I [28]. Additionally, the same antiproliferative effect occurs in AMPK-deficient cancer cells, demonstrating that the contribution of AMPK activation to the metformin antiproliferative effect might be unnecessary [29].

Hexokinase II, a glycolytic enzyme that interacts with the outer membrane of mitochondria, has higher levels in cancer cells than in normal cells [30,31]. Metformin directly interacts with glycolysis through binding at the glucose-6-phosphate (G6P) site of Hexokinase II to trigger apoptosis [29]. Another target protein of interest involves the glycerol-3-phosphate dehydrogenase of mitochondria (GPDH). Inhibition of GPDH leads to a diminished transformation of glycerol-3-phosphate to dihydroxyacetone phosphate, hence less lactate and glycerol being used in the production during gluconeogenesis [32]. As GPDH levels are increased in thyroid cancer cells, this tumorigenesis can be targeted through the interaction of metformin and the modulation of oxidative phosphorylation [33].

Apart from the above direct actions, metformin can also indirectly impact cancer cells through the systemic control of the insulin/IGF1 signaling pathways as described below.

### 3.2. Insulin- and IGF1-Dependent Mechanisms

Because cancer cells rely heavily on glucose, metformin can also act by modifying insulin and IGF1 signaling, especially in patients with type 2 diabetes [23,34]. Cells with impaired glucose metabolism or mitochondrial complex I mutations (mtDNA mutations) are also more susceptible to metformin and its derivative phenformin than wild-type cells when glucose levels are low [29].

Through the Ras/Raf/MEK/ERK and PI3K/AKT/mTORC1 (PAM) pathways, insulin and IGF1 act as growth factors for many tumors, encouraging cell survival, proliferation, and resistance to apoptosis [34,35]. Because insulin-like growth factor-binding proteins (IGFBPs) are reduced by hyperinsulinemia, the IGF1 receptor—a key factor in malignant transformation and tumor survival—is activated, increasing free IGF1 concentrations. By reducing the levels of IGF1 and insulin in the blood and suppressing the expression of their receptors, metformin interferes with this process [34,35].

### 3.3. AMPK-Dependent Mechanisms

Given that AMPK serves as a metabolic master switch—prioritizing cell survival over proliferation—its activation is widely regarded as a central mechanism behind metformin’s anticancer effects [36]. The process begins with shifts in cellular energy status; specifically, fluctuations in AMP:ATP or ADP:ATP ratios act as the primary trigger for AMPK [32]. Within this framework, Liver Kinase B1 (LKB1) acts as an indispensable mediator by directly phosphorylating AMPK [33,34]. Recent in vitro data further suggest that inositol polyphosphate multikinase (IPMK) interacts with LKB1 to amplify this response in the presence of metformin [37].

**Figure 2:**
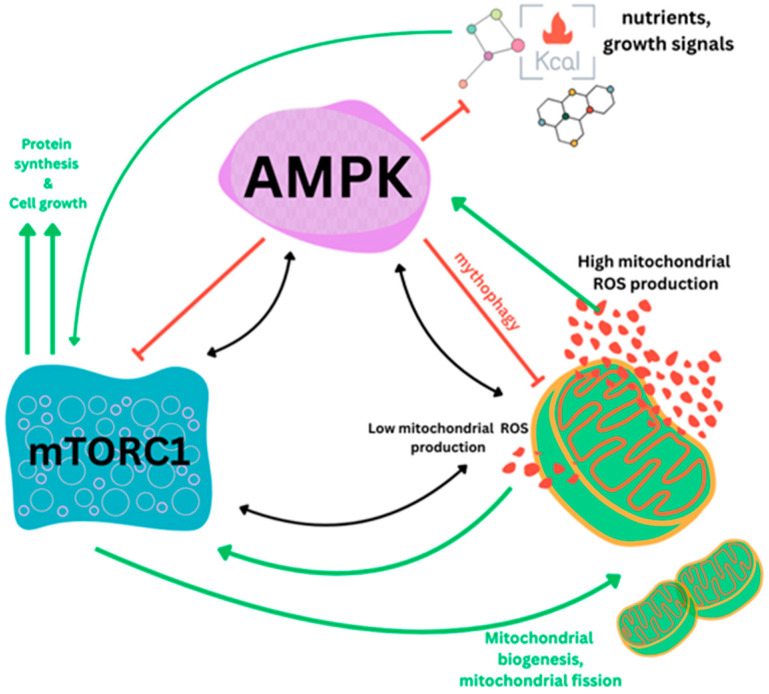
Mitochondrial stress, AMPK activation and mTORC1 modulation, interplay and feedback mechanisms. The way a cell manages its energy resources usually comes down to a constant push-and-pull between two major regulators: AMPK and mTORC1. As we see in the schematic, whenever nutrients and growth factors are around in high numbers, the balance shifts in favor of mTORC1. This essentially tells the cell that it is safe to start building—focusing on things like protein synthesis and increasing overall biomass. On the other hand, if the cell runs low on energy or starts dealing with high levels of mitochondrial ROS, AMPK steps in. It acts almost like a metabolic sensor, directly inhibiting mTORC1 so the cell can save energy instead of spending it on growth it cannot afford. This relationship is also central to how the cell maintains its ‘internal hardware.’ While mTORC1 promotes the creation of new mitochondria (biogenesis and fission) to power cell division, AMPK focuses on cleaning up the damaged ones through ULK1-mediated mitophagy. Ultimately, this ‘Yin-Yang’ interaction keeps the cell’s metabolism and mitochondrial health in a steady, homeostatic state.

Once activated, AMPK targets the tuberous sclerosis complex, phosphorylating both TSC1 (hamartin) and TSC2 (tuberin). This signaling cascade effectively shuts down Rheb GTPase, a key activator of mTORC1. Beyond this direct route, metformin exerts further control over mTORC1 through alternative pathways. For instance, it can induce the hypoxic stress-response protein REDD1 via a p53-dependent mechanism [38] or interfere with Rag GTPases, which usually drive mTORC1 activation in response to nutrient availability [39,40,41]. The interplay between nutrient availability, levels of mitochondrial oxidative stress, AMPK and mTOR1 in carcinogenesis, aging and as potential targets for metformin should therefore not be overlooked (Figure 2) [19,36,37].

Acetyl-CoA carboxylase (ACC) phosphorylation and inhibition in ovarian and prostate cancer cell lines require AMPK activation via LKB1. Gene-expression profiles are changed as a result of increased acetylation of histone and non-histone proteins [42]. Furthermore, fatty acid oxidation is increased and lipogenic enzyme levels are decreased when the ACC gene is inactivated [39].

Metformin also inhibits lipogenesis [39,43], angiogenesis [44], cytokine production [45], and CD8+ tumor-infiltrating lymphocyte infiltration [46], among other AMPK downstream effects.

### 3.4. AMPK-Independent Mechanisms

#### 3.4.1. Direct Antineoplastic Effects: Beyond the AMPK Paradigm

While AMPK is a central player, it is becoming increasingly clear that metformin’s efficacy does not rely on this pathway alone. The drug appears to operate through a dose-dependent hierarchy; at low concentrations, energy stress triggers AMPK, but at higher doses, alternative mechanisms take the lead. This suggests a redundant, multi-layered system rather than a single point of failure. Crucially, metformin can suppress tumor progression and mitigate aging markers even in cells lacking functional AMPK signaling, indicating that its systemic benefits—such as the reduction in circulating insulin—often bypass the need for local AMPK activation [47,48].

Metformin’s direct influence also extends to the tumor suppressor protein p53. In prostate cancer models, for instance, metformin significantly inhibits growth in p53-wild-type cells, whereas this effect is markedly diminished in p53-deficient environments. However, a major caveat remains: most laboratory findings rely on metformin concentrations far exceeding those found in human patients, leaving the clinical relevance of the p53-axis still open to debate [49]. Furthermore, metformin can inhibit mTORC1 by upregulating the stress-response protein REDD1 in a p53-dependent manner, effectively bypassing traditional nutrient-sensing routes [50]. Beyond these pathways, the drug actively reshapes the tumor microenvironment by downregulating pro-inflammatory and angiogenic factors such as VEGF, HIF-1α, and PDGF-B, thereby hindering the epithelial–mesenchymal transition (EMT) and limiting metastatic potential [51].

#### 3.4.2. Indirect Systemic Mediators

Metformin also combats cancer and aging by fundamentally altering the body’s systemic environment. One of its most potent indirect effects is the suppression of hepatic glucose production. By promoting the accumulation of intracellular AMP, metformin noncompetitively inhibits adenylate cyclase. This leads to a drop in cyclic AMP (cAMP) and a subsequent decrease in protein kinase A (PKA) activity—a process that effectively shuts down glucagon-mediated gluconeogenesis regardless of AMPK status [52].

Perhaps most importantly, metformin’s ability to lower circulating insulin levels remains a cornerstone of its anticancer profile. By improving insulin sensitivity and reducing hyperinsulinemia, the drug starves insulin-dependent tumors of a key growth stimulus [53,54,55]. Far from being mutually exclusive, these AMPK-independent pathways actually reinforce one another. They suggest a multi-layered mechanism where the drug’s impact is heavily dictated by the specific context: the dosage used, the drug’s distribution, and the metabolic state of the model. Because these models are so varied, there is a strong case for future research to be far more transparent. If we are to truly pin down the role of AMPK, it is vital to document intracellular metformin levels alongside the exact status of AMPK signaling—whether through genetic or pharmacological tweaks—and systemic factors like insulin and glucose levels.

### 3.5. Stress-Induced Effects

Cellular stress is a key factor that shapes the metabolic behavior of cancer cells. Metformin can make cells more susceptible to stress by restricting available energy and worsening already poor vascularization, which becomes especially important under hypoglycemic conditions [38]. When glucose levels drop, the endoplasmic reticulum (ER) activates the unfolded protein response (UPR) to prevent the accumulation of misfolded proteins [56], maintaining this adaptive response until mitochondrial respiration is restored [38,57].

**Figure 3:**
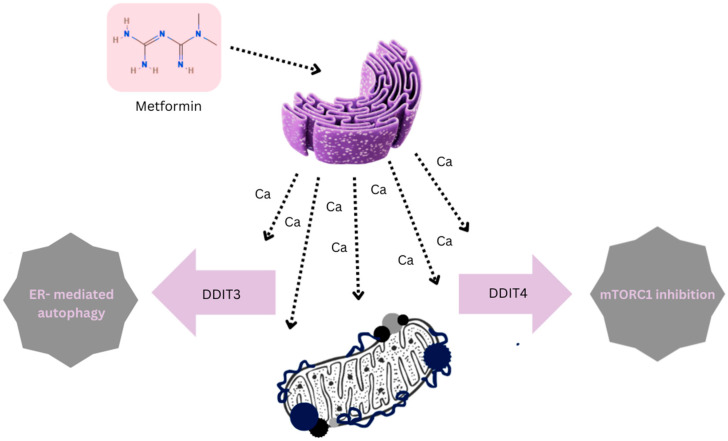
Apoptosis response through inducible factors (DDIT3 and DDIT4) induced by metformin and its direct and indirect effects on endoplasmic reticulum and mitochondria. The mechanism illustrated here centers on metformin’s ability to act as a catalyst for cellular reprogramming, beginning within the endoplasmic reticulum (ER). Rather than a localized effect, the drug initiates a systemic signaling cascade that recalibrates the delicate balance between growth and survival. By acting as a specific stimulus for the ER (the purple organelle), metformin triggers an immediate efflux of calcium ions into the cytosol. This mobilization is more than a byproduct; it serves as a critical “sentinel signal” of ER stress, alerting the cell’s internal machinery to an impending metabolic shift. This cytosolic calcium surge essentially functions as a molecular switch for the DDIT signaling axis, which serves as a sort of emergency regulatory crossroads. This involves two distinct, protein-mediated pathways: DDIT3 (GADD153), which directs the cell toward ER-mediated autophagy. This is a selective “housekeeping” process where the cell degrades its own damaged components to maintain equilibrium under duress. DDIT4 (REDD1), which acts as a metabolic checkpoint. By suppressing the mTORC1 complex, DDIT4 effectively puts the cell’s “growth engine” on hold, prioritizing resource conservation over energy-intensive proliferation. The final layer of this adaptation involves the mitochondria (the bottom organelle). As the released calcium interacts with mitochondrial respiration, it reinforces the shift toward a stress-adapted phenotype. Ultimately, the schematic reveals how metformin forces a total reallocation of cellular resources, abandoning an anabolic growth trajectory in favor of a resilient, survival-oriented state.

Evidence from tumor cell cultures and xenograft studies shows that severe ER stress can lead to mitochondrial blebbing, largely due to excessive Ca^2+^ influx from the overstressed ER. This, in turn, triggers apoptosis through stress-responsive mediators such as DDIT4 and DDIT3 (Figure 3) [38,58,59]. DDIT4 contributes to mTORC1 inhibition and thereby slows tumor cell proliferation, while DDIT3 plays a central role in initiating ER-mediated apoptotic pathways.

### 3.6. Autophagy, Apoptosis Induction, and Cell Cycle Arrest

**Figure 4:**
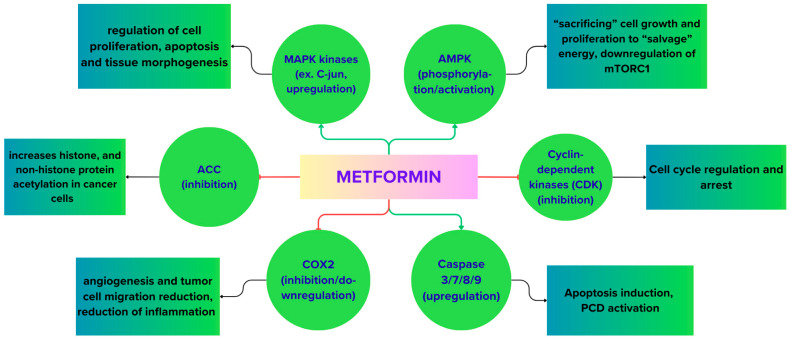
Some of the target enzymes for the metformin’s antineoplastic activity. The diagram illustrates some of the key metformin mechanisms of action and signaling molecules and enzymes it affects. By negatively regulating enzymatic targets such as Acetyl-CoA carboxylase (ACC), CDK, and pro-inflammatory singling molecules such as cyclooxygenase 2 (COX2), it increases histone and non-histone acetylation in transformed cells; reduces inflammation; angiogenesis and cell migration as well as arresting the cell cycle in key checkpoints. Conversely, by activating key crucial metabolism and signaling “balancing” enzymes such as Mitogen-Activated Protein Kinases, AMPK and the pro-apoptotic caspases (3/7/8/9), it shifts the metabolism towards conservative status–preventing growth, proliferation, and inducing programed cell death and apoptosis. Essentially, under metformin’s influence, the cells are encouraged to prioritize careful energy utilization and survival instead of rapid growth.

Autophagy is a double-edged sword in cancer biology because, on one side, it offers a survival signal for cancer cells under metabolic stress but, on the other side, it can act as an initiating factor for apoptosis. The differences in these adaptations are mediated by AMPK, which acts as a “thermostat” at the cellular level that detects metabolic stress and triggers the warning system for protective mechanisms [60,61]. The activation of AMPK directly initiates autophagy by regulating the autophagy-start signaling machinery, including mitophagy (Figure 2), which helped in the clearance of damaged mitochondria and consequently led to cell homeostasis. Apoptosis and autophagy are two prominent forms of programmed cell death (PCD) in normal and tumor cells [43,48,59]. Since metformin has the potential to trigger PCD, often through AMPK or enzymatic pathways, it is increasingly being explored as an adjunct in cancer therapy, where the focus would be on encouraging stressed tumor cells towards death rather than survival and enhancing the efficacy of existing treatments [Figure 4].

A clear example comes from the work of Takahashi et al., who found that metformin caused cell cycle arrest at both G1 and G2/M phases in Ishikawa endometrial cancer cells by upregulating the cyclin-dependent kinase inhibitor p21 (CDKN1A) [62]. At higher doses (10 mM), metformin also activated caspase-dependent apoptosis (CASP3/7, -8, -9). Interestingly, the apoptosis response was reduced when the autophagy regulator Beclin1 (BECN1) was inhibited [63].

Variations in the tumor suppressor gene TP53 impact heavily upon tumor characteristics. Approximately 50% of various cancers exhibit irregularities of the p53 protein [63,64]. In the case of p53 proficient colon cancer xenografts, the growth of the tumor was unaffected by metformin, though apoptosis occurred. However, in the case of p53-deficient colon cancer xenografts, the growth of the tumor was inhibited along with apoptosis triggered by metformin [65].

In breast cancer models, Al-Zaidan et al. found that metformin at a concentration of 10 mM resulted in G1 arrest, reduced cell viability, and apoptosis in 72 h [66]. Similarly, Li et al. reported that metformin caused G2/M phase accumulation in osteosarcoma cells both in vitro and in vivo, potentially by reactivating NAC-suppressed JNK/c-Jun signaling. This stress-dependent pathway ties metformin to autophagy, apoptosis, and cell cycle arrest in these cells [67]. However, a critical translational caveat is that these phenotypic changes—such as robust induction of apoptosis and autophagy—are predominantly documented at suprapharmacological concentrations (10 mM). These doses are approximately 100 to 1000 times higher than the steady-state plasma levels of 1–5 μM typically achieved in patients [68]. Consequently, while these studies elucidate the maximal biological signaling potential of metformin, they may overstate its potency as a monotherapy in a clinical setting.

In acute myeloid leukemia SKM-1 cells, metformin induced G0/G1 arrest through an AMPK-dependent mechanism [69]. Treatment decreased CDK4 and Cyclin D1 levels, increased p53 expression, and showed a clear dose-dependent timeline of antiproliferative effects: 1 mM peaked at 72 h, 5–10 mM at 48 h, and 15–20 mM at 24 h. Despite being higher than typical therapeutic concentrations, these doses did not lead to lactic acidosis or other severe adverse effects, which is a promising observation [70,71].

### 3.7. Metformin in Therapy Combinations and Dose-Dependent Clinical Effects

We are seeing a real shift in how metformin is being tested—specifically, how it behaves at lower, safer doses when paired with other drugs. It turns out that metformin is not just a “stand-alone” treatment. Instead, it seems to act more like a sensitizer, making chemotherapy, hormone therapies, or even DNA-targeting agents work a bit harder [72].

The big takeaway here is that at these low doses, metformin does not actually kill cells on its own. Its real value is in lowering the “apoptotic threshold.” For instance, when combined with hormone treatments, it can actually tinker with gene expression and halt cell proliferation in ways that the drugs could not manage alone. There is even some evidence that it helps shield the heart from the toxic side effects of certain antibiotics. That said, it is not a universal fix; the synergy with drugs like cisplatin, for example, is still pretty hit-or-miss [72,73].

Take the work by Rafaela Erices and her team. They found something quite telling: ovarian cancer cells became far more vulnerable to carboplatin when metformin was added at doses similar to what a diabetic patient would take [73]. On its own, metformin at that level did almost nothing to stop cell growth. This really illustrates the point that metformin’s future in oncology is not as a “miracle pill” on its own, but rather a strategic add-on. By keeping the doses safe and tolerable, we might finally make resistant tumors more responsive to the standard tools we already have.

There are many pathways through which the synergy can occur in metformin. The AMPK/mTOR can be mainly implicated when co-administered in hormone-modulating therapy, while the role of HIF-1, P-gp, and MRP1 approached suppression can be crucial when combined with anti-metabolites [72]. Of late, there has been a study of the synergy of metformin when combined with the phytochemicals quercetin and resveratrol. When co-administered with quercetin, the combination of metformin has been proven to target the reduced viability, migration, and invasiveness of prostate cancer cells (PC-3 and LNCaP). However, the combination of metformin and resveratrol has demonstrated the role of proliferation through PI3K/Akt downregulation, AMPK phosphorylation, and the modulation of mTOR when combined together [74]. The role of nanovectorization has been shown to further enhance the bioavailability and synergistic effects of metformin and phytochemicals.

Berberine, which has antidiabetic and antineoplastic actions similar to metformin’s and is a phytochemical, also has the shared mechanistic targets of AMPK activation and JNK/c-Jun, CDK4, mTOR, and WNT pathways [75,76]. Clinical trials involving the co-administration of the two in metabolic syndrome patients indicate their combined effectiveness over the two drugs separately [77], though there is less information regarding their interaction in cancer research.

The dosing regimen of metformin has a direct impact on its medicinal value. The medication demonstrates varying efficiencies for different types of cancer and other diseases like polycystic ovary syndrome and cardiovascular diseases [77,78]. The dosage of metformin when used to promote longevity and healthspan will be less compared to its dosages in metabolic and cancer treatment applications (Table 1).

Aging has been linked to various biological characteristics: genomic instability, telomere shortening, epigenetics, proteostasis failure, disrupted nutrient sensing pathways, mitochondriopathies, senescent cells, depletion of stem cell pools, dysregulation of intercellular interaction, inflammation, and dysbiosis. Most of the above factors can be traced to the existence of chronic inflammation and oxidative stress in the body, which can cause the development of the tumor microenvironment [79,80,81].

Patients with both T2DM and cancer often share risk factors such as age, sex, obesity, limited physical activity, and suboptimal diet [82]. Hyperglycemia worsens oxidative stress and increases advanced glycation end products (AGEs), adding to genotoxic burden and elevating cancer risk [83,84]. By modulating glucose and insulin metabolism and reducing IGF-1 levels, metformin offers protective benefits—particularly among older individuals with T2DM [85]. In one analysis, the adjusted hazard ratio (HR) for cancer incidence was 0.68 (95% CI 0.51–0.90), although cancer mortality did not change. Interestingly, concurrent daily aspirin use (100 mg) was associated with higher cancer incidence in the same cohort [86].

Systemic inflammation, immunosenescence, and the activation of JNK in the elderly also cause vulnerability to cancer [87,88,89]. Metformin has been found to reduce the production of various factors that cause inflammation, such as IL-6 and NF-Κb [90]. This also reduces the production of PGE2 through the suppression of COX-2 and the expressions of Snail and IL-6 due to its anti-migratory and anti-angiogenic actions [91,92,93,94].

In cancer, among the key factors responsible for the activation of cancer stem cell-like properties, is the dysregulation of Wnt-catenin signaling. The abnormal activation of this signaling pathway is responsible for the continuous proliferation of cells, resistance to treatments, and changes in the tumor microenvironment [95]. For example, it has been shown that metformin, through the activation of AMPK and the subsequent inhibition of mTOR, indirectly inhibits Wnt signaling. Consequently, the transcriptional activity of catenin is inhibited [96,97].

**Figure 5:**
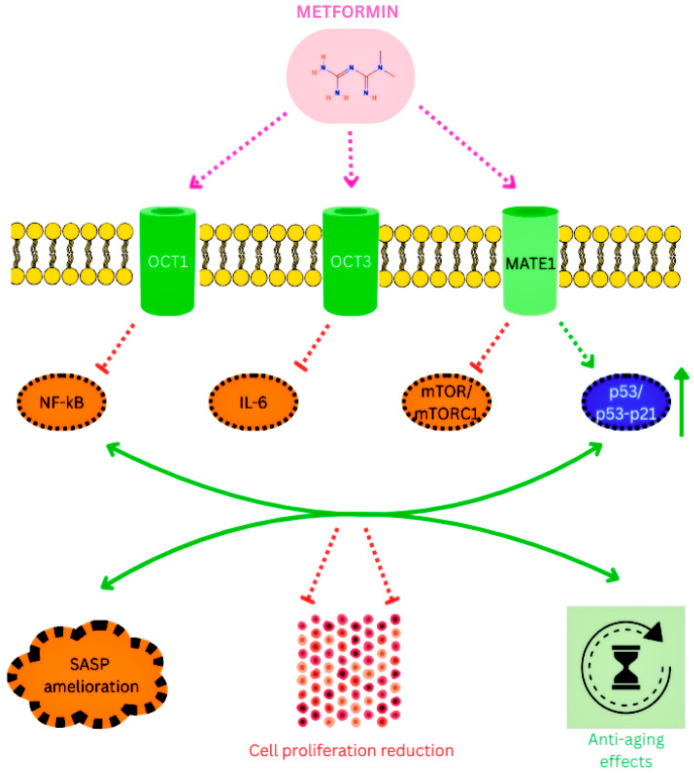
Metformin-driven regulation of cellular senescence and longevity pathways. This is the diagram representing a multitargeted mechanism for metformin in attenuating age-related phenotypes and reducing the pro-inflammatory cellular milieu. Metformin gets into the target cells driven by the organic cation transporters, mainly OCT1, OCT3 and MATE1, crucial for its intracellular accumulation. Intracellular metformin acts as a regulator and/or a potent antagonist of major inflammatory molecules: NF-κB and IL6, through which it seems to quell the chronic, low-grade inflammation that is a common feature of biological aging. Cell proliferation is significantly reduced as a result of mTOR/mTORC1 suppression and positive regulation of the p53-p21 system. Furthermore, p53/p21 axis upregulation supports genomic stability and a balanced cell cycle. These molecular interactions work together to produce positive effects essential in cancer cell’s proliferation reduction and increased cellular lifespan/health span, mainly through SASP amelioration and p53 activation. By lowering the Senescence-Associated Secretory Phenotype (SASP), metformin stops senescent cells from sending out inflammatory signals to unaffected cells.

However, it should be noted that most of the evidence for these effects is based on in vitro studies, which are primarily carried out using metformin concentrations that are several times higher than the pharmacologically relevant levels, and thus, not attainable in patients [98]. Hence, the implications of metformin on the Wnt pathway in human cancers are yet to be determined. Moreover, the relationship between Wnt signaling and senescence in cancer is complex. On the one hand, the inhibition of senescence, associated inflammation, and the factors of SASP can inhibit the signals that promote tumors in the microenvironment, but on the other hand, the over-inhibition of senescence can, in theory, inhibit its cancer-inhibiting effects, especially in the very early stages of tumor formation (Figure 5) [99,100]. These points highlight the complexities involved in preclinical research and the need for careful interpretation of the results and their validation through rigorous, context, dependent studies [101,102,103].

Together, these evidence points demonstrate the different biological outcomes of Wnt and SASP pathway modulations in cancer, and therefore these evidence points cannot be merely translated to other biological or pathological processes, especially aging, where the biological roles of these pathways are quite different [95,98,102].

Regarding aging and age-related diseases, the Wnt signaling pathway and cellular senescence are two key elements that clearly determine the pathophysiological process. Therefore, the stem cell population dynamics, the capacity of the tissue to regenerate, and the susceptibility to the degenerative diseases are the common biological effects of the dysfunction of the Wnt pathway, which has been linked to the natural aging process. Under these conditions, it has been proposed that a controlled downregulation of the ab-errant Wnt signaling may favor tissue preservation and ultimately lifespan extension, [96,104] which is something metformin may possibly achieve based on the available data.

Preclinical studies have indicated that metformin could modulate age and age-related pathways primarily by mitigating mitochondrial oxidative stress, enhancing metabolic efficiency, and counteracting chronic low-grade inflammation triggered by the accumulation of senescent cells. The modulation of certain aspects of the Senescence-Associated Secretory Phenotype, therefore, has been hypothesized to be one of the mechanisms by which metformin could mitigate age-related inflammatory issues and slow the onset of functional decline [105]. However, the bulk of the evidence available to date has been obtained from animal and/or cell-based studies, and there is a complete lack of human validation.

In contrast to cancer, where senescence can be either protective or detrimental, depending on the context, reducing persistent SASP (Senescence-Associated Secretory Phenotype) signaling in aged tissues is thought to be, for the most part, a positive process [90,106]. The extent to which this occurs, and when, and in which tissues, is still unclear. To assess the effect of metformin on senescence burden and SASP activity, a long-term human study will be needed to determine if metformin modulates these processes at a clinically relevant dose.

## 4. Clinical Evidence and Translational Implications

The clinical evidence on the anticancer and anti-aging effects of metformin remains heterogeneous and has been derived largely from observational studies [48,63,66,72,80,81,100]. Despite the fact that a large number of retrospective studies have shown that patients with type 2 diabetes taking metformin have lower incidence of cancer or improved survival rates, these observations should be interpreted cautiously [22,24,26,87]. They could be influenced by confounding by indication, immortal time bias, and marked metabolic heterogeneity (Table 2).

Moreover, the existing evidence indicates a high degree of variability of clinical outcomes depending on the type of cancer. A more consistent positive association has been observed in colorectal, breast, and hepatocellular cancers, whereas the findings in lung, prostate, and pancreatic cancers have been mostly neutral or inconsistent [16,20,33,70,74,93,105]. It is also important to note that the randomized controlled trials conducted so far have not been able to demonstrate a definite anticancer effect of metformin in non-diabetic patients, thus underlining the difficulty of extrapolating preclinical data to the clinical setting [47,50,103].

From a translational perspective, the clinical relevance of metformin appears to be highly context-dependent. For instance, systemic metabolic disorders, drug accumulation in particular tissues, genetic predisposition of tumors, and combination therapies are likely to influence therapeutic responses [27,35]. Another issue is that most of the mechanistic information has been derived from in vitro studies using suprapharmacological concentrations of metformin that are substantially higher than the plasma concentrations that can be achieved clinically [106].

## 5. Future Directions

Trying to repurpose metformin from a standard diabetes pill into a multi-purpose tool for cancer and aging is not just a matter of running more trials. It actually demands a total rethink of research logic. The singular approach does not promise as much as researching synergism and tailored, individualized doses to specific metabolic phenotypes of a patient.

Perhaps the most persistent issue in the field is the ‘dose-translation gap,’ a discrepancy that undermines much of available in vitro data. Much of in vitro data comes from lab tests using concentrations that are far higher from what you would actually find in human plasma (the typical 10–50 µM range). If our models are ever going to mean anything in a clinic, we have to prioritize doses that actually make sense for the human body. Validating pathways like p53 or Wnt at concentrations humans can actually handle safely is the objectively best way forward.

Additionally, currently available data would suggest that metformin’s real strength is that of a strategic “assistant.” By nudging the “apoptotic threshold” of cancer cells, it could make tumors more vulnerable to standard chemo like Cisplatin—maybe even helping to break through drug resistance. There is also a strong case for its role in immunotherapy, helping to “clean up” the metabolic mess in the tumor microenvironment. Combining it with senolytics or natural compounds like Resveratrol is an exciting frontier, more so if these attempts eventually hold up in human trials.

Also, to reduce or evade side effects like lactic acidosis or stomach issues, we may need a smarter delivery system. Crucially, utilizing nanoparticles as targeted vehicles allows for a more concentrated release of metformin where it is needed most, potentially mitigating the common off-target effects and gastrointestinal distress that currently limit its use. Its effects on B12 levels and VO max (possible reduction) in older adults also warrant further research.

It is important to address the polymorphisms in population which are of interest here. We simply cannot expect uniform results from a population with such diverse metabolic profiles. Future research requires more precision, tailoring and individualization-oriented approach. We need to find the markers—LKB1 status, OCT1 levels, or even gut bacteria—that will “tell us” who will actually respond.

Concerning its life-extending prospective effects: while preclinical data is encouraging, we must acknowledge that murine models are, at best, an approximation of human physiological responses. We desperately need long-term studies like the TAME trial as that is the only proper way to prove if metformin can truly push back age-related diseases and actually extend the human healthspan.

## 6. Conclusions

It is impossible to overlook how much metformin actually interferes with the core cellular pathways behind both cancer and aging. But we need to stay grounded: moving these lab results into a real clinical setting is a massive uphill battle. We can see the drug hitting targets like inflammation and mitochondria, but we are still quite far from pinning down how metabolic control and cancer onset really talk to each other.

Perhaps the biggest problem facing researchers and the first thing they tend to gloss over is the “dose translation gap.” A massive percentage of our data comes from in vitro studies with unrealistic and massive doses of this stuff. This is not even close to the human body and is completely unrealistic in someone who is not even diabetic in the first place. The “sweet spot” of dosage and timing in a completely healthy human is not just the next step in this process; it is the most pressing issue for the next ten years of study.

If we look at it from a molecular standpoint, the intersection of AMPK and mTOR provides a good lead. In “cleaning up” the chronic inflammation that cancer cells and aging cells feed off of, metformin effectively “cleans up” the mess. Of course, we have to stop referring to it as a “miracle pill,” however. Realistically speaking, it is likely not as effective as a standalone drug. It is more likely to have an impact as a smart, synergistic “add-on” to what we are currently using. To actually get there, we need to move past the idea that one size fits all. We have to have human trials that examine the unique metabolic signature of an individual. It is only then will we be able to tell if the promise we are seeing in a lab setting will translate into a real-life victory.

## Figures and Tables

**Table 1:** Reported metformin doses in preclinical and clinical studies.

Context of Use	Model/Study Type	Typical Dose Range	Reported Effect
Lifespan/Healthspan	*C. elegans*, *Drosophila*; rodent models	10–100 μM (in medium); 50–300 mg/kg/day (oral)	Increased lifespan via AMPK activation; reduced ROS; improved healthspan; delayed tumorigenesis [72,73,74]
Cancer research	In vitro cancer cell lines (breast, colon, leukemia, osteosarcoma); xenograft mouse models; clinical studies (adjuvant use)	1–20 mM; 100–300 mg/kg/day; 500–2000 mg/day (oral)	Autophagy and apoptosis induction; cell cycle arrest; tumor growth inhibition; improved therapy response and survival outcomes [62,65,66,67,69]
Metabolic/Classical Use	T2DM patients	500–2000 mg/day (oral)	Glycemic control; reduced insulin resistance [70,71]

**Table 2:** Examples of discussed clinical evidence and translational implications of metformin in oncology and aging.

Clinical Evidence (Cancer/Aging Outcomes)	Essential Mechanism	Study Type	Translational Significance	Reference
Reduced overall cancer risk in T2DM patients (Scottish population study)	Systemic insulin/IGF-1 reduction; indirect mTOR inhibition	Population-based cohort	Supports chemopreventive potential in metabolically dysregulated populations	[8]
31% reduction in overall tumor incidence and 34% reduction in cancer-related mortality in diabetic patients	AMPK activation; mTOR suppression; metabolic reprogramming	Meta-analysis of diabetic cohorts	Rationale for repurposing as adjunct anticancer therapy in T2DM patients	[13]
Decreased incidence of gastrointestinal cancers (colon, liver, pancreas)	Reduced hyperinsulinemia; IGF-1 signaling attenuation	Observational diabetic cohorts	May justify targeted prevention strategies in high-risk GI cancer patients with T2DM	[16]
Improved overall survival in metformin users (dose-dependent effect)	Energy stress induction; systemic metabolic modulation	Observational diabetic cohorts	Suggests dose optimization could enhance oncologic outcomes	[12]
No significant survival benefit in colorectal cancer with T2DM (HR 1.06; 95% CI 0.80–1.40)	Heterogeneous response; context-dependent metabolic effects	CRC patients with T2DM	Highlights need for biomarker-driven patient stratification	[17]
Most pronounced survival benefit in breast cancer; moderate in colorectal and prostate cancers (meta-analysis of 80 studies)	Combined AMPK/mTOR and insulin-lowering mechanisms	Meta-analysis (80 observational studies); aggregated populations	Indicates tumor-type-specific responsiveness and supports precision and targeted oncology approaches	[18]
Reduced cancer incidence (adjusted HR 0.68; 95% CI 0.51–0.90); no change in cancer mortality	Glucose lowering; reduced oxidative stress and IGF-1	T2DM cohort	Suggests preventive potential, rather than therapeutic clinical application	[86]
Reduced inflammatory markers (IL-6, NF-κB) independent of diabetes status	Anti-inflammatory AMPK/NF-κB modulation	Human study	Reinforces potential role in addressing inflammation-driven tumorigenesis and aging	[90]

## Data Availability

No new data were created or analyzed in this study. Data sharing is not applicable to this article.
